# Simultaneous Disruption of the Pubic Symphysis and Sacroiliac Joint during Vaginal Birth

**DOI:** 10.1155/2015/812132

**Published:** 2015-05-20

**Authors:** Hakan Çıçek, H. Levent Keskın, Ümit Tuhanıoğlu, Kasım Kiliçarslan, Hasan Ulaş Oğur

**Affiliations:** ^1^Department of Orthopedics, Adana Numune Education and Research Hospital, 1358 Adana, Turkey; ^2^Department of Obstetrics and Gynecology, Atatürk Education Research Hospital, Ankara, Turkey; ^3^Department of Orthopedics, Atatürk Education Research Hospital, Ankara, Turkey

## Abstract

*Background*. Puerperal diastasis of the pubic symphysis is a rare intrapartum complication. This report presents the case of a woman who experienced synchronous pubic symphysis and sacroiliac joint separations induced by vaginal delivery. *Case*. A 32-year-old woman (gravida 2, parity 2) with an uncomplicated prenatal course developed acute-onset anterior pubic pain during vaginal delivery. The pain persisted postpartum and was exacerbated by leg movement. Physical and radiographic examinations showed a pubic symphyseal separation of 2.4 cm, accompanied by a 10 mm disruption of the left sacroiliac joint. The patient was treated conservatively with pain-relief medication; bed rest, mostly in the left lateral decubitus position; closed reduction and application of a pelvic binder; use of a walker; and physical therapy. *Conclusion*. The patient responded to conservative management. She was essentially pain-free and regained movement and ambulation by 12 weeks postpartum.

## 1. Introduction

Separation of the pubic symphysis in association with pregnancy, labor, and delivery is a rare and frequently unrecognized complication [1]. The reported incidence of this condition has varied widely, ranging from about 1/569 to 1/30,000 deliveries [1–5].

Progressive lordosis is a characteristic feature of normal pregnancy. The increased lumbar lordosis caused by the weight and position of the developing fetus shifts the center of gravity backward over the lower extremities and increases the stress on the lumbosacral junction and sacroiliac joints. These anatomical changes present mechanical challenges to the musculoskeletal system during pregnancy, including a predisposition to pelvic laxity and joint mobility that may contribute to the alteration of posture. The bones and ligaments of the pelvis adapt remarkably to pregnancy, and slight increases in the mobility of the sacroiliac, sacrococcygeal, and pubic joints are considered normal and necessary for childbirth.

Progesterone, relaxin, and estrogen cause the connective tissues of the ligaments of the pubic symphysis and sacroiliac joints to relax during pregnancy, allowing the joints to respond to mechanical stress [1, 6, 7]. Relaxin targets the pubic symphysis, causing changes in the extracellular matrix that may be important in the modification and relaxation of this site during pregnancy [6, 7]. However, the relationship between hormone levels and joint laxity in pregnancy remains unclear. Increases in peripheral-joint laxity during pregnancy do not correlate with serum relaxin, estradiol, or progesterone levels [8].

The extent of symphyseal changes during pregnancy and delivery may vary significantly among individuals. Physiological widening is minor and asymptomatic. Peripartum ligamentous relaxation with moderate asymptomatic widening of these joints is physiological and occurs regularly [1, 4]. This response begins to regress immediately after parturition and regression is complete within 6 months. However, separation of more than 10 mm may occur rarely and is generally symptomatic [1]. The sacroiliac joint exhibits marked mobility at term and this displacement, which is greatest in the dorsal lithotomy position, may increase the diameter of the pelvic outlet by 1.5–2.0 cm. However, some women experience much greater degrees of pelvic-girdle relaxation. Anterior separation of the pubic symphysis of more than 2.5 cm causes progressive injury to the posterior pelvic ring, including disruption of the sacroiliac joint or sacral fracture [4].

Although many case series and case reports have discussed peripartum pubic symphysis separation [1–3], few have examined the disruption of the pubic symphysis with accompanying posterior pelvic-arch instability after natural childbirth [4, 9]. In this report, we present the case of a patient suffering from symptomatic symphysis diastasis with accompanying disruption of the left sacroiliac joint associated with vaginal delivery. This patient was treated conservatively. The importance of our case lies in the synchronous disruption of the two joints, rather than the degree of each separation. We also present a brief literature review to accompany the case presentation.

## 2. Case Presentation

A 32-year-old, 79 kg woman (gravida: 2, parity: 2) was admitted to our hospital for anterior groin, lower-back, and hip pain associated with leg movements and difficulty in walking. She had delivered an infant weighing about 3500 g 4 days previously in a different hospital. The delivery occurred at 38 gestational weeks after an uncomplicated prenatal course and followed 7-8 hours of continuous second-stage labor attended by a physician. An episiotomy was performed, but no medication was administered to induce or augment labor. The patient stated that uterine fundal pressure had been applied during the second stage of labor. During and immediately after delivery, the patient felt a shearing pain in the area of the pubic symphysis and reported that she was unable to move from the table. She was discharged from the hospital on the first postpartum day with suggestions of bed rest and the use of paracetamol.

The patient's first delivery was 12 years earlier and was uneventful. Her history included no chronic medical problem, previous surgery, or trauma. The patient provided written informed consent to the use of her data in this case report.

**Figure 1:**
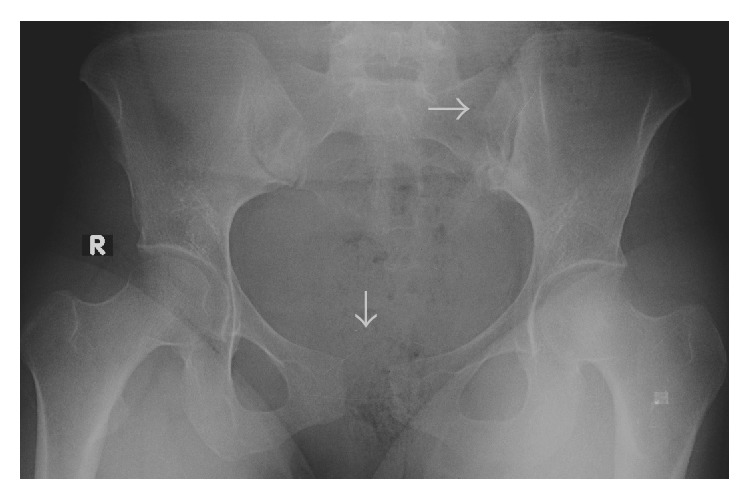
Anteroposterior radiograph of the pelvis demonstrating gross disruption of the pubic symphysis, with a separation of 2.4 cm and disruption of the left sacroiliac joint.

**Figure 2:**
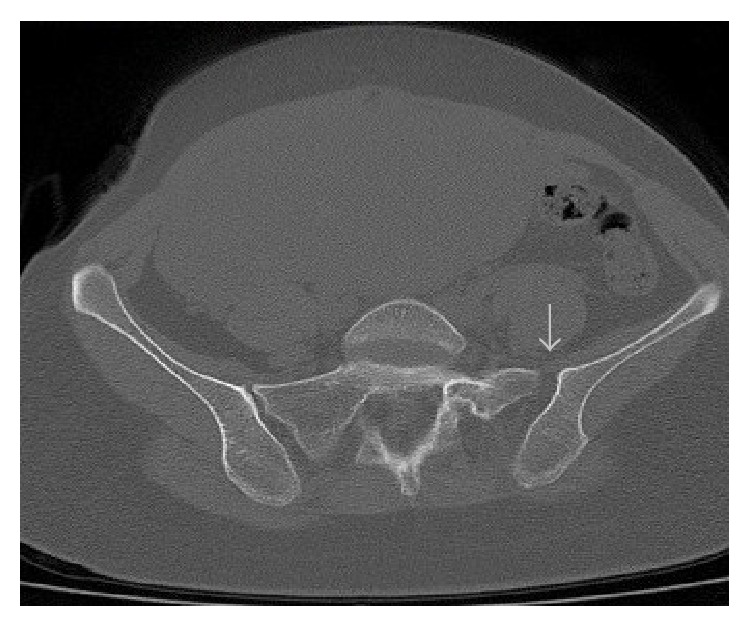
Computed tomography images demonstrating widening of the left anterior sacroiliac joint.

Gynecological examination revealed no marked genital bleeding and was unremarkable, except for the presence of bilateral localized edema on the labia majora. The episiotomy incision was intact and clear. The neuromuscular examination was normal and the patient had no difficulty during urination. After the initial examination, we requested a consultation from the orthopedic department. During the orthopedic examination, the patient complained of pain in the pubic symphysis and sacroiliac joints and radiating down the left thigh. The lower extremities, especially the left side, were externally rotated and the patient was unable to walk normally. She had a waddling gait and was unable to fully activate the left hip. The examination revealed moderate point tenderness at the pubic symphysis and a 2–2.5 cm gap between the superolateral edges of the pubic bones. An external-rotation stress test identified mild to moderate instability and significant pain, especially in the area of the left iliac crest. An anteroposterior radiograph of the pelvis was taken while the patient was in a supine position on the delivery table and revealed a 2.4 cm diastasis of the pubic symphysis and an approximately 1 mm separation of the left sacroiliac joint (Figure 1). A computed tomography (CT) scan of the pelvic joints revealed similar findings (Figure 2).

This pelvic dislocation was diagnosed as type II anteroposterior compression (APC-II). Although two joints were affected, the disruptions were not severe and we observed no other indication of pelvic instability. The patient was thus treated conservatively with pain medication, bed rest, a pelvic binder, a walker, and physical therapy. A well-padded pelvic binder was applied while the patient was in the left lateral decubitus position and she was restricted to complete bed rest, mostly in this position. Paracetamol (3 × 500 mg/d) was administered for 3 weeks to relieve pain and enoxaparin sodium (40 mg/d) was administered subcutaneously for 4 weeks due to the increased risk of thromboembolism caused by immobilization. The patient was discharged on the second day after the application of the pelvic binder with the ability to void spontaneously. She was maintained at home on a regimen of strict bed rest in the lateral decubitus position for 6 weeks. The pain decreased gradually during this time. Physical therapy consisting of in-bed isometric exercises for both lower extremities was initiated in the first posttraumatic week and active joint motions were allowed at 3 weeks. At 6 weeks postpartum, the pelvic binder was removed and walker-assisted ambulation that relieved up to 25% of the weight on the lower extremities was allowed. The patient regained the ability to walk unassisted and experienced no symptoms during resting or mobilization in the third month postpartum.

**Figure 3:**
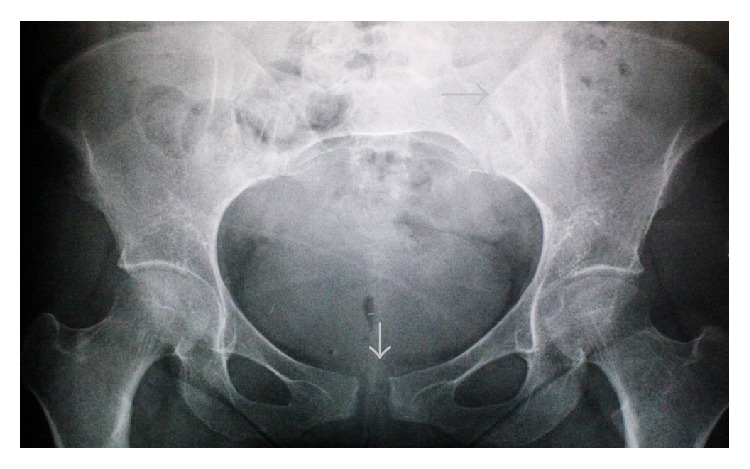
Anteroposterior radiograph of the pelvis demonstrating reduction of the pubic symphysis and left sacroiliac joint at 6 months postpartum.

The patient was followed up with anteroposterior radiography of the pelvis at 3-week intervals and was asymptomatic and healthy in the sixth posttraumatic month. Follow-up radiographs indicated the reduction of the pubic symphysis and left sacroiliac joint, with a gap of about 1 cm remaining in the pubic symphysis (Figure 3).

## 3. Discussion

Peripartum rupture of the pelvic joints is a rare complication with underestimated consequences. About 1.1% of patients with pelvic and acetabular fractures caused by high-energy trauma are pregnant women [10]. The pubic symphysis is most frequently affected by such trauma. The slight widening is common during pregnancy with a mean 7-8 mm separation. This widening is more pronounced in multiparas than in primigravidas. In asymptomatic patients, the mean gap is 4.8 mm. Rarely, the separation is greater than 10 mm; such cases are usually considered pathological [1]. In symptomatic cases, the mean gap is 20 mm (range: 10–35 mm). Separations as great as 120 mm have been reported and the sacroiliac joints become affected when the separation exceeds 40 mm [1].

In symptomatic separation of the pubic symphysis or one of the sacroiliac synchondroses, the patient may complain of a sharp and immediate onset of severe pain in the symphyseal region that may be accompanied by an audible “crack” and may extend posteriorly into the sacroiliac joint region, radiating down the thighs and legs during labor or delivery [3, 4]. Such separations are associated with tenderness of the pubic symphysis, considerable swelling, disability, marked interference with movement, and, occasionally, bladder dysfunction. A persistent loss of reduction can cause substantial disability in postpartum women. All persistent symptoms are related to the sacroiliac disruption [9].

Palpation of the pubic symphysis may reveal a gap associated with edema and hematoma of the overlying soft tissue, and vaginal examination may reveal a palpable separation. Pain usually occurs with movement, especially when the patient stands or walks [1].

Clinical history, presenting symptoms, and response to therapy are sufficient for the diagnosis of this type of injury, although the documentation of symphyseal separation by radiography or ultrasound is frequently used to confirm the diagnosis [5]. APC-II trauma should be considered in the diagnosis of patients who experience the acute onset of pain during delivery that does not improve postpartum [11].

The etiology of symptomatic symphyseal separation has not been fully elucidated. Numerous potential etiological factors have been implicated in the separation of the pubic symphysis, including difficult parturition or precipitous labor; cephalopelvic disproportion, macrosomia, shoulder dystocia, or abnormal presentation; multiparity; previous trauma or excessive force applied to the pelvic ring, excessive abduction of the thighs during delivery, or difficult operative vaginal delivery using forceps or vacuum extraction (especially in cases of fetal/pelvic disproportion); preexisting abnormality due to congenital dysplasia, osteomalacia, chondromalacia, rickets, tuberculosis, and arthritis; and excessive hormone-related softening of the ligaments during pregnancy [1, 5, 9, 11, 12].

Maternal age, parity, and fetal weight play no clear role in the development of obstetrical related symphysiolysis. Rapid descent of the presenting part of the fetus in the second stage of labor, however, is a common feature [2]. The incidence of symptomatic separation appears to be decreasing over time, as many difficult vaginal deliveries and operative instrumental deliveries are increasingly replaced by Cesarean section [1].

Ruptures of the pubic symphysis appear to result from the extraordinary forceful descent of the fetal head against the pelvic ring, although the extraordinary forces required for such ruptures do not occur during normal labor or delivery [1].

The application of uterine fundal pressure is a controversial maneuver that aims to reduce the duration of second-stage labor. Although no confirmed benefit has been documented and some adverse events have been reported in association with its use, this maneuver is used widely [13, 14]. Uterine fundal pressure was applied to our patient during the second stage of labor. We believe that the use of inappropriate and uncontrolled force, such as mentioned above, on the pelvic girdle during this maneuver may have caused the disruption of the joints and contributed to the separation of the pelvic girdle in our patient. The use of uterine fundal pressure in the second stage of labor is thus a risk factor for pelvic disruption.

Separation of the pelvic ring during pregnancy and delivery is normal. Symptomatic patients with no sign of gross instability and a separation of less than 1 cm may be observed carefully and serially. However, patients exhibiting instability or a separation of more than 1 cm, or who experience diastasis symptoms such as pain, bladder dysfunction, or ambulatory difficulty, require treatment and follow-up. There is no consensus on the best treatment for pregnancy-related pubic symphyseal or the other pelvic-girdle joint separation [15]; conservative and aggressive treatments are currently used [1].

Most treatments of ruptured pubic symphyses consist of nonsurgical management, including analgesia, activity restriction, rest in the lateral decubitus position, an appropriately fitted pelvic binding, ambulation devices, and physical therapy [16]. Conservative treatment followed by early mobilization is adequate for separation of the pubic symphysis or sacroiliac joint [3]. This method usually results in the alleviation of symptoms in as little as 2 days and complete functional recovery within 4 to 8 weeks [1, 5, 9].

Surgery is occasionally indicated, particularly when nonsurgical treatment is unsuccessful. Operative treatment has been described in selected cases showing inadequate reduction, recurrent diastasis, or persistent symptoms [9, 15, 17]. An operative approach may be necessary to preserve the integrity of the pubic symphyseal joint when diastasis exceeds 4.0 cm [9]. External or internal fixation with plates and screws or cerclage wire on the superior pubic rami is the treatment of choice to maintain stability while the ligaments heal [17–19]. The aggressive treatment of severe pubic symphysis separation with fixation results in the patient's early ability to ambulate, void, and care for herself and her baby [15].

Symphyseal ruptures may indicate posterior pelvic-arch instability and require reduction and stable fixation. These injuries result in unstable pelvic disruption and may correspond to traumatic APC-II or APC-III or Tile type B or C pelvic injuries. Patients should be managed in the same manner used for trauma patients with pelvic fractures, including vigilant monitoring of hemodynamic status and aggressive resuscitation, appropriate diagnostic imaging studies, and timely operative reduction and fixation of the pelvis.

Open reduction and internal anterior-plate fixation (ORIF) of the pubic symphysis with simultaneous posterior percutaneous screw fixation of the sacroiliac joints is a treatment option for synchronous symphyseal and sacroiliac joint disruption [4, 20].

More than 50% of separations recur in subsequent pregnancies, although the risks of recurrence remain poorly defined [21]. Previous symphyseal separation should not significantly alter the management of subsequent pregnancies, and conservative therapy is recommended for any recurrence of symptoms. The mode of delivery for subsequent pregnancies should be discussed with the mother, due to the previous traumatic experience and the mother's potential fear of recurrence [21]. Vaginal delivery may be proposed in productive discussions with the patient that include consideration of prevention and therapeutic options. Cesarean delivery may also be considered [22].

Pregnancy and associated physiological changes, as well as risk factors such as the application of uterine fundal pressure during pregnancy and labor, may cause damage to the pelvic ring that simultaneously affects the anterior and posterior joints. Pelvic-ring instability can be managed and treated by symptomatic medical treatment and compression, or alternatively by surgery.
